# The Enhancement Origin of Antioxidant Property of Carboxylated Lignin Isolated from Herbaceous Biomass Using the Maleic Acid Hydrotropic Fractionation

**DOI:** 10.3390/ijms25179257

**Published:** 2024-08-27

**Authors:** Chen Su, Xiu Wang, Yongjun Deng, Douyong Min, Guigan Fang, Chen Huang

**Affiliations:** 1 Guangxi Key Laboratory of Clean Pulp & Papermaking and Pollution Control, College of Light Industry and Food Engineering, Guangxi University, Nanning 530004, China; chensu@njfu.edu.cn (C.S.); mindouyong@gxu.edu.cn (D.M.); 2 Jiangsu Co-Innovation Center of Efficient Processing and Utilization of Forest Resources, Institute of Chemical Industry and Forest Products, Chinese Academy of Forestry, Nanjing 210042, China; yongjun_deng@126.com (Y.D.);; 3 Key Laboratory of Polymer Chemistry and Physics of Ministry of Education, School of Materials Science and Engineering, Peking University, Beijing 100871, China

**Keywords:** lignin, antioxidant property, maleic acid, carboxyl groups, structure-activity relationship

## Abstract

Lignin is endowed with antioxidant activity due to its diverse chemical structure. It is necessary to explore the relationship between antioxidant activity and the chemical structure of the lignin to develop its high-value utilization. Herein, we employed maleic acid (MA) as a hydrotropic agent to preferably isolate the lignin from distinct herbaceous sources (wheat straw and switchgrass) under atmospheric pressure conditions. The resultant acid hydrotropic lignin (AHL) isolated from wheat straw exhibited high radical scavenging rates, up to 98% toward DPPH and 94% toward ABTS. Further investigations indicated that during the MA hydrotropic fractionation (MAHF) process, lignin was carboxylated by MA at γ-OH of the side-chain, providing additional antioxidant activity from the carboxy group. It was also found that the radical scavenging rate of AHL has a positive correlation with carboxyl, phenolic hydroxyl contents, and the S–G (syringyl–guaiacyl) ratio, which could be realized by increasing the MAHF severity. Overall, this work underlies the enhancement origin of the antioxidant property of lignin, which will facilitate its application in biological fields as an efficient, cheap, and renewable antioxidant additive.

## 1. Introduction

Lignin, the second most abundant natural polymer and a primary component of the cell wall in lignocellulosic biomass, is the main available renewable resource based on aromatic units [1,2,3,4]. Due to its structural complexity and multi-functions, lignin draws great attention and hope as a bio-based material in various fields [5,6]. In recent years, many researchers have reported that lignin has distinguished bioactivity, and its performance is closely associated with its chemical structures [7,8]. Meanwhile, lignin from herbaceous biomass with unique chemical structures displays excellent antioxidant activity, which exhibits utilization potentiality in healthcare fields [9,10,11].

Xie et al. reported that the lignin extracted from olive leaves had stronger antioxidation than that from olive fruit toward O_2_^−^ radicals due to the higher phenolic hydroxyl (-OH) content [12]. Jiang et al. evaluated the radical scavenging abilities of lignin from rice straw and found that the lignin with higher phenolic -OH content showed better radical scavenging ability [13]. Many publications [13,14,15] have reported that the radical scavenging capacity of lignin could be improved effectively with higher phenolic -OH content. However, during the lignin isolation process, besides the change in phenolic -OH, other structural properties are also significantly altered, such as the carboxyl group (-COOH) and the S–G ratio. The effect of phenolic -OH on the antioxidant activity of lignin has been demonstrated, but the influence of the other structural changes on the antioxidant activity is less understood. The hardship of accurately controlling the content changes in these two structures in lignin is the key issue that has perplexed the researchers in establishing a relationship between these structures and the antioxidant activity of lignin. Therefore, it is crucial to find a proper isolation method to control the -COOH content and S–G ratio in lignin.

Traditional lignin separation methods, including acid or alkaline [16,17], steam explosion [18], and organosolv [19], primarily aim to extract cellulose by dissolving hemicelluloses or lignin. These processes are conducted at severe fractionation conditions, leading to the formation of condensed lignin, which is unsuitable for its value-added applications. Recently, reductive catalytic fractionation [20], deep eutectic solvent [21], and ionic-liquid-based systems [22] have been burgeoning ways to isolate the biomass; however, these approaches have not fully resolved the issues related to chemical recovery, toxicity, and regulated structural variation. Consequently, the effective fraction of lignin from lignocellulosic biomass continues to pose significant challenges.

Maleic acid hydrotropic fractionation (MAHF) has been developed in our lab and demonstrated its advantages for the rapid fractionation of herbaceous biomass at atmospheric pressure and low temperatures [23,24]. Meanwhile, several advantages were demystified for our present work: (1) the carboxylation and S–G ratio of lignin can be regulated by fractionation conditions [25,26]; (2) lignin can be rapidly fractionated at low temperatures under atmospheric pressure and short durations [26,27]; and (3) acid hydrotropic lignin (AHL) separation could be achieved by diluting the fractionation liquor below the minimal hydrotropic concentration (MHC) of MA [24]. Given these advantages, MAHF was used to separate lignin from herbaceous biomass in the present study.

Herein, this study uniquely unveils the enhanced mechanism of antioxidant properties for AHL isolated from herbaceous biomass using MAHF. Unlike some reported studies that primarily focused on the impact of phenolic -OH content on the antioxidant activity of lignin, our research delves deeper into the contributions of the -COOH group and the S–G ratio. By controlling the conditions of the MAHF, we successfully modulated the chemical structures of the lignin, particularly the content of -COOH and -OH groups, as well as the S–G ratio, leading to the increased scavenging rates of AHL towards DPPH and ABTS radicals. The radical scavenging rates of AHL isolated from wheat straw (L-M60W) were high, at up to 98% toward DPPH and 94% toward ABTS. This breakthrough offers a promising avenue for developing sustainable, high-performance antioxidant additives, with broad implications for the biopharmaceutical industry and beyond.

## 2. Results and Discussion

### 2.1. The Physical Properties of AHL and MWL

**Figure 1:**
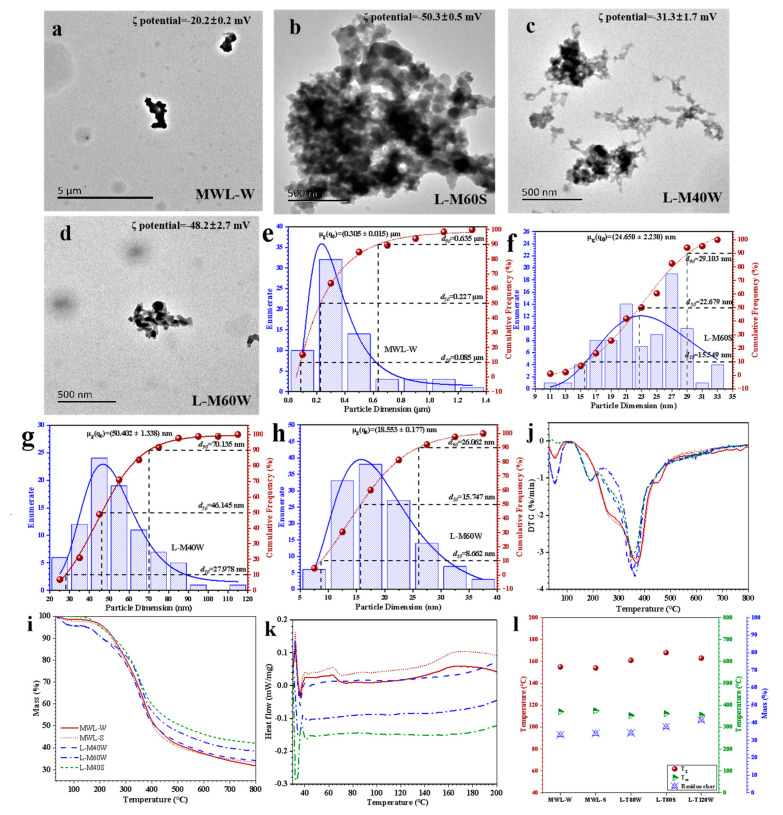
TEM images of MWL–W and AHL: (**a**) MWL–W; (**b**) L–M60S; (**c**) L–M40W; (**d**) L–M60W; particle size distribution, particle geometric mean, and ζ potential of MWL and MAHL: (**e**) MWL–W; (**f**) L–M60S; (**g**) L–M40W; (**h**) L–M60W; thermal stability of MWL and AHL: (**i**) TG, (**j**) DTG, (**k**) DSC, and (**l**) residue char.

**Figure 2:**
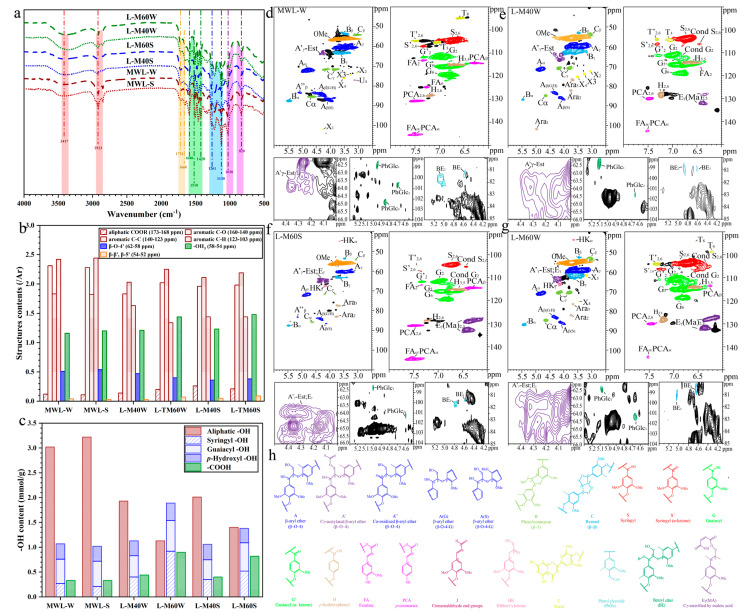
Chemical structure analysis of AHL and MWL: (**a**) FT-IR spectra; (**b**) quantitative ^13^C NMR information; (**c**) quantitative ^31^P NMR information; (**d**–**h**) 2D HSQC NMR and main substructures: (**d**) MWL-W, (**e**) L-M40W, (**f**) L-M60S, (**g**) L-M60W, (**h**) the main structures of lignin.

The morphologies and ζ potential of AHL are shown in Figure 1a–d and Figure S1. We can see that the particle size of most AHL was below 100 nm. With the increasing fractionation severity, good sphericity and decreasing diameter distribution of AHL were achieved, such as for L-M60W for which the measured geometric mean was the lowest of 18 nm (Figure 2h). The reason was that the S–G ratio increased as the fractionation severity intensified, as 2D NMR affirmed (Figure 2e–g), and the non-covalent π–π interaction between S unit lignin was stronger than that of G unit lignin [28], thus leading to a denser packing of lignin molecules during AHL formation and resulting in a smaller average size of AHL. Moreover, all AHL were negatively charged, exhibiting ζ-potential values lower than −20 mV. The negative surface charge was ascribed to the highly abundant phenolic -OH and -COOH of the AHL surface, which prevented lignin from agglomeration during microsphere formation and stabilized lignin in a colloidal dispersion due to the electric double-layer repulsion [29,30]. In comparison to AHL, the MWL-W featured irregular and rod-like agglomerates, with a larger particle size exceeding 300 nm (Figure 1e).

Compared with the MWL, the AHL showed a lower average molecular weight (*Mw*), especially for the L-M60W and L-M60S, with an *Mw* of approximately 4600 mol/g (Table 1). When increasing the fractionation conditions, the decrease in *Mw* was severe, while the *Mw* of L-M60S was just 60~70% of L-M40S due to the depolymerization reaction during MAHF [31]. Moreover, with increasing severity, the PDI (polydispersity index) decreased, and the *Mw* of L-M60S was only half that of MWL-S, suggesting that increasing the fractionation condition resulted in more uniform lignin.

The thermal properties of MWL and AHL were investigated by Thermo Gravimetric Analysis (TGA) and Differential Scanning Calorimetry (DSC), as shown in Figure 1i–l. The major weight loss stage occurred between 200 and 300 °C and 300 and 400 °C, which was responsible for the decomposition of hemicellulose remains and lignin, respectively. The highest maximum temperature of weight loss (T*_m_*) appeared at 371 °C for MWL-W because of the highest content of β-O-4′ linkages and *Mw*, which are the two main factors affecting T*_m_* [32,33]. Compared to the MWL, the T*_m_* of all AHL decreased, indicating that AHL could be more easily degraded. The reason for this was that MAHF cleaved the β-O-4′ linkages and depolymerized lignin to reduce *Mw*. The T*_m_* of L-M60W was much greater than that of L-M40W, which was because L-M60W was obtained from severe fractionation conditions, and more thermostable C-C bonds between lignin were formed during the repolymerization reaction and thus elevated the T*_m_* [25].

Lignin with a stable thermal character is the primary property for application as a bioactive material in various fields. As shown in Figure 1k,l, the glass transition temperature (T*_g_*) of MWL-W and MWL-S was around 155 °C, whereas the T*_g_* of AHL was much higher than that of MWL. Under similar fractionation severity, L-M60W had a much higher *Mw* and condensed degree than those of L-M60S, which resulted in significantly increased T*_g_* of L-M60W. Compared to L-M40W and L-T120W, the T*_g_* of these two lignin samples increased with increasing fractionation conditions, though the *Mw* of L-T120W decreased by the depolymerization reaction. However, the condensation degree of L-M60W was much greater than that of L-M40W and thus led L-M60W to an elevated T*_g_* due to the improved thermostable C-C bonds.

### 2.2. Chemical Structures of AHL and MWL

As shown in Figure 2a, the main FT-IR absorption lines of samples were assigned according to the previous reports [34,35]. In the carbonyl stretching region, intensive peaks at 1725 cm^−1^ were assigned to the acetyl group of the lignin, which was produced by the esterification during the MAHF, which increased with increasing severity, i.e., the intensity of L-M60W was stronger than that of L-M40W. The absorption at 1261 cm^−1^ was attributed to the stretching vibration of C-O in the G unit; meanwhile, the peak intensity could be weakened stepwise by increased fractionation conditions. This result was likely due to the G unit lignin being condensed during the severe fractionation conditions.

**Figure 3:**
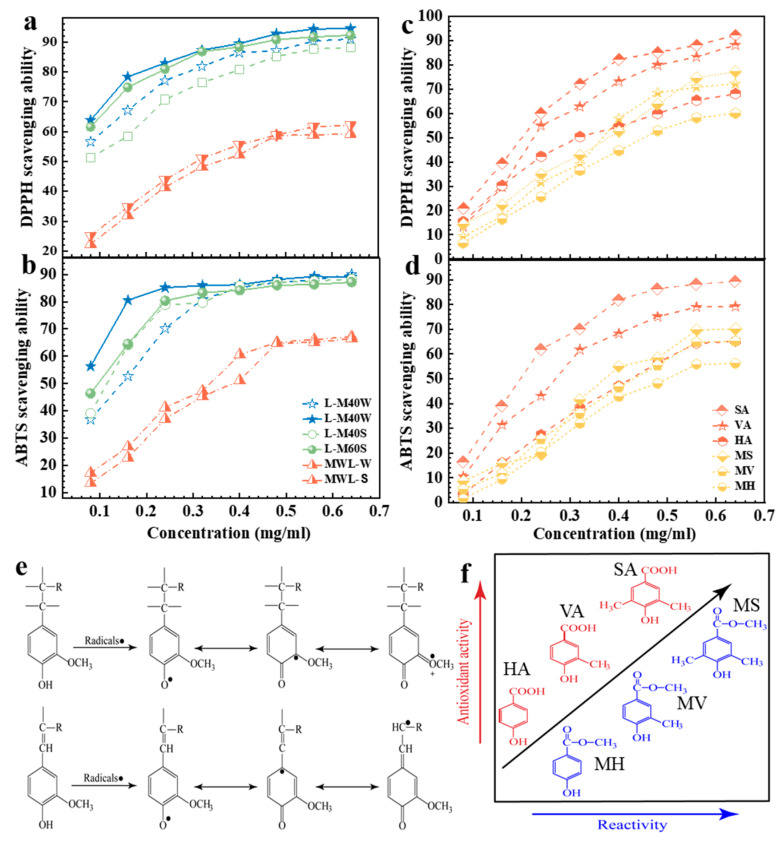
The scavenging ability of (**a**) MWL, (**b**) AHL, and (**c**,**d**) lignin monomers toward DPPH and ABTS radicals; (**e**) trapping and stabilization mechanism of radicals by lignin; (**f**) schematic of the relationship of different lignin monomers between reactivity and antioxidant activity.

The structural changes in AHL and MWL were further investigated by quantitative ^13^C NMR (Figure S2a), and the calculation method was based on previous works [36,37]. The aromatic region in the ^13^C NMR spectra from δ 160.0 to 103.0 ppm could be divided into three broad regions: oxygenated aromatic carbons (δ 160.0–140.0 ppm), aromatic carbon–carbon (C-C) (δ 140.0–123.0 ppm), and aromatic methine carbons (δ 123.0–103.0 ppm), as shown in Figure 3b. The amounts of aliphatic -COOR of L-M40W, L-M60W, and L-M60S were 0.14/Ar, 0.20/Ar, and 0.26/Ar, respectively (Figure 2b). The results were in agreement with the 2D NMR spectra, i.e., the MA_γ_ intensity of AHL obtained from switchgrass was stronger than that from wheat straw, indicating that the γ-OH in AHL from switchgrass was more prone to being esterified by MA. However, the contents of the C-C structure in all kinds of AHL increased with intensified fractionation conditions. The C-C content in L-M60W was much higher than that in L-M60S, suggesting that both types of lignin were condensed by forming C-C bonds [25]. Moreover, AHL from wheat straw (AHL-W) was more easily condensed than that from switchgrass (AHL-S), under the same fractionation severity, which was consistent with the 2D NMR analysis (Figure 2e–g, Table 1).

To determine the variation in the -OH in the lignin preparations after MAHF,^31^P-NMR spectra of MWL and AHL were used to determine the -OH contents, as shown in Figure 2c and Figure S2b [38,39]. MAHF resulted in a dramatic reduction in the aliphatic -OH content, which decreased from 3.2 to 1.1 mmol/g (MWL-S vs. L-M60S, Figure 3c), suggesting that more aliphatic -OH were substituted by MA with increasing reaction severity [24]. Furthermore, the content of phenolic -OH in AHL was significantly increased, indicating that a fairly large number of β-O-4′ linkages were cleaved during MHAF. Additionally, no significant change in *p*-hydroxyl -OH was observed in AHL, suggesting that MAHF delignification did not result in the demethoxylation reaction as reported by other AHF methods [40,41]. In comparison, the -COOH content of all AHL was much greater than that of MWL, with the reason being that the lignin esterification (carboxylated) by MA could improve -COOH content in all AHL.

To better understand the structure features of lignin, the selected AHL and MWL were analyzed by 2D HSQC NMR spectroscopy. The obtained spectra are shown in Figure 2d–g and Figure S3, and the assigned peaks are illustrated in Table S1, where the quantification of lignin substructures (Table 1) expressed as per 100 aromatic lignin units (/100Ar) are based on published works [42,43,44].

Compared with MWL-W, in the L-M40W (Figure 2d,e), a small change in β-O-4′ linkages can be observed, which were reduced to less than 6/100 Ar. Under severe conditions, the content of β-O-4′ was significantly decreased, with only 26/100 Ar of L-M60W; however, the β−5′ and β−β’ content increased with increasing fractionation conditions. A similar tendency could also be found in AHL-S. The stronger cross-signals at δ_C_/δ_H_ 128.5/6,23, 132.3/6.39 and 63.5/(3.83, 4.30) ppm related to the typical characteristics of lignin were evidence of esterification (carboxylated) by MA at the γ-OH position to form E_γ_(MA), as reported by our earlier study [23]. The esterification (carboxylation) of AHL increased with the enhanced MAHF severity, and the contours of E_γ_(MA)_2,3_ from L-M60W at 128.5/6,23, 132.3/6.39 ppm were significantly expanded in comparison with L-M40W. The signal intensity of E_γ_(MA)_2,3_ from L-M60S was much stronger than that of L-M60W, which was consistent with the results in Table 1 (i.e., 46% and 39%). Additionally, the signals (δ_C_/δ_H_ 44.5/3.70 ppm, HKα; δ_C_/δ_H_ 67.1/4.20 ppm, HK_γ_) from ketone of the Hibbert route were identified in the side-chain region of L-M60S and L-M60W, suggesting that the ketone of the Hibbert route was the main degraded pathway for β-O-4′ linkage cleavage and the recondensation reaction during MAHF [26]. 

As listed in Table 1, the S–G ratio of AHL was improved after MAHF, especially for AHL-W. Comparing the L-M40W and L-M60W, the S–G ratio of L-M60W increased to 0.5, suggesting that most of the G unit lignin of AHL-W was dissolved in the MAHF process with increasing severity [24]. The signal from Condensed S_2_._6_ can be found at δ_C_/δ_H_ 105.5/6.7 ppm in Figure 2e–g. Compared with L-M40W, the abundance of Cond S_2.6_ in L-M60W increased from 2/100 Ar to 9/100 Ar of L-M40W. Moreover, the same trend could also be observed in Cond G_2_ subunits, as when increasing the fractionation conditions, Cond G_2_ increased from 3/100 of L-M40S to17/100 Ar of L-M60S.

The LCC linkages are known as phenyl glycoside (PhGlc) and benzyl ether (BE) [45], which are identified in the zoomed-in 2D NMR spectra of Figure 2d–g. Three correlation signals in MWL-W spectra were shown at δ_C_/δ_H_ 98.6/5.02 ppm (PhGlc1), 100.6/4.70 ppm (PhGlc2), and 101.9/4.77 ppm (PhGlc3) [46,47]. However, some signals of PhGlc disappeared in both types of AHL due to the glycoside bonds breaking partially in the acid hydrotrope. Regarding the BE linkages, two types of these structures were observed in lignin preparations: (1) C1 linkages between the α-position of lignin and primary OH groups of carbohydrates (BE_1_, δ_C_/δ_H_ 81–80/4.7–4.5 ppm); (2) C2 linkages between the α-position of lignin and secondary OH groups of carbohydrates (BE_2_, δ_C_/δ_H_ 81–80/5.1–4.9 ppm) [48]. The abundance of BE decreased as fractionation severity increased because of the unstable characteristic of the acidity. Overall, the LCC linkages decreased significantly during the MAHF, and abundant signals of LCC linkages decreased or perished, which was helpful for the separation of purified lignin without carbohydrates and beneficial for enhancing the antioxidant [2].

### 2.3. Assessment of Radical Scavenging Ability of AHL and MWL

The antioxidant ability of MWL and AHL toward the DPPH and ABTS is shown in Figure 3a,b. All AHL presented higher antioxidant ability than MWL toward both radicals, which could be further improved by increasing the concentration of AHL. When increasing the fractionation condition, the scavenging ability of AHL increased, i.e., L-M60W achieved the highest elimination rate of 94% and 90% toward DPPH and ABTS, respectively (Figure 3a,b). The radical scavenging ability against DPPH of lignin was better than that against ABTS due to the different elimination reactions of lignin against the two radicals. Based on our previous work [49,50], we knew that the chemical structures of lignin were changed during the MAHF process, especially for varying contents of phenolic -OH, which results in the radical scavenging capacity of lignin occurring to the variation. Overall, the lignin from wheat straw (AHLs-W) had better antioxidant ability than that from switchgrass (AHLs-S). Compared to the MWL or AHLs-S, the significantly changeable structures of AHLs-W were carboxylation and the S–G ratio, which processes the higher -COOH content and S–G ratio better than AHLs-S. However, the effect of carboxyl groups and the S–G ratio on the antioxidant ability of lignin is not well understood [51].

To determine how the structures of lignin affect its free radical scavenging ability, the antioxidant ability of different lignin monomer models was tested, including syringic acid (SA) and methyl syringate (MS), vanillic acid (VA) and methyl vanillate (MV), and *p*-hydroxybenzoic acid (HA) and methyl 4-hydroxybenzoate (MH), as shown in Figure 3c,d. The lignin phenolic acids (SA, VA, and HA) presented much greater antioxidant ability than the phenolic esters (MS, MV, and MH), suggesting that the -COOH outperformed the ester group at increasing the antioxidant ability of lignin.

As listed in Table 1, L-M60W and L-M60S were from the same fractionation condition; however, the S–G ratio of L-M60W was two times higher than that of L-M60S, and the scavenging rate of L-M60W was higher than that of L-M60S for both radicals. As shown in Figure 3d, when increasing the -OCH_3_ content, the lignin monomer displayed a higher radical elimination ability, i.e., 92% scavenging rate of SA and 68% of HA toward DPPH at 0.64 mg/mL. This suggested that S units of AHL played the main role in free-radical scavenging, and the antioxidant ability of lignin increased with the -OCH_3_ content of the lignin unit. This phenomenon is likely due to the S units usually having two methoxy groups, which could activate the aromatic ring through an electron-withdrawing–inducing effect to trap more radicals [13,52,53], the schematic of which is illustrated in Figure 3e,f. Comparatively, G and H units only carried one and zero methoxy groups, respectively, providing no antioxidant ability enhancement for AHL [38]. Therefore, this indicated that lignin with a higher S–G ratio and more -COOH groups could display excellent antioxidative capacity, which is the necessary structure for lignin to act as a natural antioxidant.

## 3. Materials and Methods

### 3.1. Materials

The Department of Biological Systems Engineering, University of Wisconsin-Madison provided the switchgrass. The air-dried wheat straw was harvested from Canada. The switchgrass and wheat straw were Wiley-milled with a 1-cm mesh size screen. The materials that passed through the screen were used for this study. The anhydrous MA (ACS reagent grade), DPPH, and ABTS diammonium salts (ACS high-performance liquid chromatography (HPLC) grade) were purchased from Sigma-Aldrich (St. Louis, MO, USA). The other chemical reagents were of analytical grade and purchased from Macklin Biochemical Co., Ltd. (Shanghai, China).

### 3.2. Separation of Acid Hydrotropic Lignin (AHL) from Gramineae Using Maleic Acid Hydrotropic Fractionation (MAHF) and Preparation of Milled Wood Lignin (MWL)

**Figure 4:**
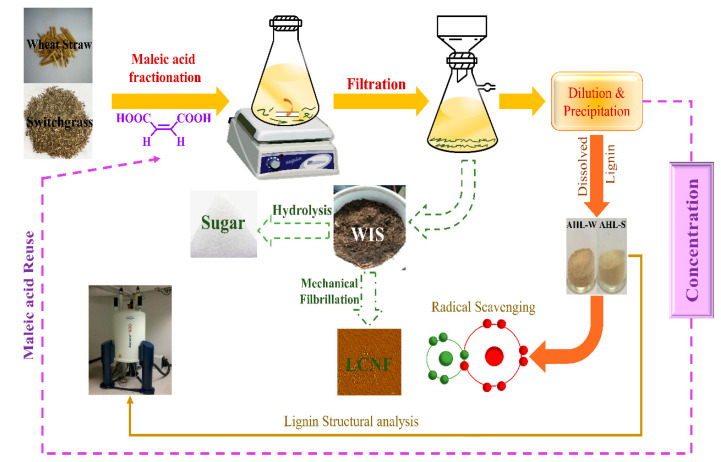
Schematic flow of herbaceous biomass (wheat straw and switchgrass) fractionation. Processes with dashed lines were not carried out in this work.

Wheat straw and switchgrass were both fractionated by MA with a concentration ranging from 40 to 60 wt%, and the reaction temperature and time varied from 80 to 120 °C and 30 to 120 min, respectively, as shown in Figure 4. The aqueous MA solutions with different concentrations were prepared in a glass flask by solubilizing the desired amounts of MA in deionized (DI) water, while the weight was fixed at 150 g. Each flask was placed on a magnetic agitator with temperature control (C-MAG HS7DS1, IKA, Wilmington, NC, USA) to facilitate the dissolution of MA. Ten grams of the oven-dried sample (wheat straw or switchgrass) were added to 150 g of the prepared MA solution under constant stirring at the designated temperature and time. The water-insoluble solids (WISs) were washed with DI water until the filtrate was diluted to 10 wt% to precipitate lignin. After centrifugation, the lignin was dialyzed in DI water for a week and then freeze-dried and named AHL. These runs were designated as M*xx*T*yy*t*zz*, with *xx*, *yy*, and *zz* representing the MA concentration in wt%, the fractionation temperature in °C, and the duration in min, as shown in Table 2.

For the preparation of milled wood lignin (MWL), air-dried wheat straw/switchgrass was milled to pass through 30 mesh in a Wiley mill (Model No. 2, Thomas Scientific, Swedesboro, NJ, USA). Then, the dried powder was milled in a vibratory ball mill (PM100, Retsch, Haan, Germany) for 12 h, followed by the MWL preparation procedure described in previous work [24,54].

### 3.3. Characterization of AHL and MWL

Acetylated lignin was prepared for GPC analyses according to previous work [13]. Briefly, 0.1 g of the freeze-dried AHL was dissolved in 2 mL of pyridine-acetic anhydride (1:1, *v*/*v*) solution, and the solution was placed on a temperature shaker (in dark condition) at 150 rpm and 40 °C for 72 h. The mixture was then added dropwise into 100 mL of ice-cold DI water with continuous stirring to precipitate the lignin acetate. The lignin acetate was collected and washed with DI water and freeze-dried. The various acetylated AHL was dissolved in tetrahydrofuran (THF, HPLC grade) with a concentration of 1 mg/mL, and analyzed by LC-20A GPC (Shimadzu, Japan) using an organic size-exclusion chromatography column (KF-804, 300 mm × 8 mm i.d., 7 μm), calibrated with polystyrene standards (peak Mw of 1000, 2000, 3000, 10,000, and 40,000 g/mol). The column was run at 40 °C and eluted with THF at a flow rate of 1 mL/min.

The morphologies of AHL and MWL were characterized using transmission electron microscopy (TEM, JEM-2100, JEOL, Tokyo, Japan). FT-IR spectra of lignin preparations were recorded by Spectrum Two (PerkinElmer, Buckinghamshire, UK) with a universal attenuated-total-reflection (ATR) probe. All samples were freeze-dried before analyses. ^13^C NMR, ^31^P NMR, and 2D ^1^H-^13^C heterogeneous single quantum correlation (HSQC) NMR of lignin samples were analyzed using a Bruker AVANCE 600 MHz spectrometer equipped with a 5 mm BBO probe using an inverse gated proton decoupling sequence. For ^13^C NMR analysis, 100 mg of the preparation was dissolved in 0.5 mL DMSO-*d*_6_ solution and then added to 40 μL 0.01 M of chromium (III) acetylacetonate. In ^31^P NMR analysis, 20 mg of nonacetylated lignin was dissolved in 0.5 mL of anhydrous pyridine-*d*_5_/CDCl_3_ (1.6/1, *v*/*v*). Then, 100 μL of cyclohexanol (11.02 mg/mL, internal standard) and 100 μL of chromium (III) acetylacetonate (5 mg/mL, relaxation regent), prepared using anhydrous pyridine-*d*_5_/CDCl_3_ solution, were mixed with the lignin solution and added to 60 μL of the phosphorylating regent (2-chloro-4,4,5,5-tetramethyl-1,2,3-dioxaphospholane), which was shaken at room temperature for 30 min and then immediately tested. For the 2D HSQC NMR experiment, 70 mg of lignin was dissolved in 0.5 mL of dimethyl sulfoxide (DMSO)-*d*_6_ as described previously [55]. The number of various lignin substructures was calculated from the NMR spectra, according to the reported works [56,57,58].

Thermogravimetric analysis (TGA) and differential scanning calorimetric (DSC) were used to analyze the thermal properties of all lignin samples. Approximately 5~10 mg of the sample was weighed and placed in the TGA instrument (TG 209 F1 libra, Netzsch, German), heating at a rate of 10 K/min from 30 °C to 800 °C under a dry nitrogen (N_2_) atmosphere. For the DSC test, approximately 5~10 mg of the sample was accurately weighed and then encapsulated in an aluminum pan. The pan was placed in a DSC tester (DSC 214 Polyma, Netzsch, German), and the heating rate was fixed at 10 K/min. The whole testing process was performed under an N_2_ atmosphere, and the flow rate was 40 mL/min. Three cycles occurred in the whole testing process: (1) samples were heated from 30 °C to 200 °C (cycle 1); (2) samples were cooled down to 50 °C and kept at this temperature for 20 min (cycle 2); and (3) samples were then reheated to 200 °C (cycle 3). The plot obtained from this second heating run showed the glass transition temperature (T*_g_*) as a step transition.

### 3.4. DPPH and ABTS Radicals Scavenging Ability

The DPPH and ABTS radical scavenging assay of lignin preparations was performed using a spectrophotometric method according to previous publications [49,59]. Briefly, the lignin preparations were dissolved in dimethyl sulfoxide/water (9/1, *v*/*v*) with different concentrations (0.08~0.64 mg/mL), and then tested with DPPH and ABTS radical solutions. The amount of remaining DPPH and ABTS in the assays was analyzed by a spectrophotometer (Model 8453, Agilent Technologies, Palo Alto, CA, USA) at 517 nm and 734 nm, respectively.

## 4. Conclusions

This study demonstrated that MAHF was an effective method to isolate lignin with enhanced radial scavenging capacity from herbaceous biomass. AHL was esterified (carboxylated) by MA at the γ-OH of the lignin side-chain, and the carboxylation degree, S–G ratio, phenolic -OH, and -COOH contents of AHL were all highly dependent on the MAHF severity. Under the same MAHF severity, the thus-obtained AHL-W had higher *Mw* with a wider distribution and higher scavenging ability compared to AHL-S. More C-C bonds were formed in AHL-W due to the repolymerization reaction, consequently resulting in better thermostability with higher T*_m_* (362 °C) and T*_g_* (168 °C). To prove the effect of the S–G ratio and -COOH group on antioxidant activities, lignin from different species (wheat straw and switchgrass) was chosen for comparison. We found that AHLs-W had a higher S–G ratio and -COOH content than AHLs-S under the same fractionation conditions. Moreover, L-M60W and L-M60S under fractionation conditions of 60 wt% of MA, 110 °C, and 60 min reaction temperature and times had a higher esterification degree (more -COOH group) and S–G ratio. The radical scavenging rates of L-M60W were high, with up to 98% toward DPPH and 94% toward ABTS. This suggested that the S–G ratio, in conjunction with -COOH, played a synergistic role in determining the comprehensive antioxidant capacity of lignin. The significance of our findings is underscored by the potential for developing sustainable and high-performance antioxidant additives from renewable herbaceous biomass, which paved the way for elaborately isolating lignin from renewable herbaceous biomass for wide utilization in pharmaceutical fields as antioxidant additives.

## Figures and Tables

**Table 1:** Amount of lignin substructure and LCC linkages along with the molecular weight (*Mw*) of MWL and AHL samples (/100 Ar).

Lignin Samples	MWL-W	MWL-S	L-M40W	L-M60W	L-M40S	L-M60S
*Mw*	12,817	13,659	8798	4664	7915	4592
*Mn*	4589	4971	4176	3174	4067	3207
*Mw/Mn*	2.8	2.7	2.1	1.5	1.9	1.4
Interunit linkages						
β−O−4′	55.2	48.1	51.8	26.1	43.7	28.7
β−5′	6.7	5.5	4.4	5.3	3.1	4.4
β−β′	6.0	6.3	5.3	8.7	1.8	2.5
Condensed degree	18.7	19.7	18.6	34.9	10.1	19.3
γ-esterification	11.5	12.3	16.0	38.5	18.2	46.0
HKα	-	-	-	8.2	2.4	4.2
Aromatic units						
S	39	33	49	58	34	40
Cond S	-	-	2	9	1	8
G	58	63	48	39	63	57
Cond G	-	-	3	21	3	17
H	3	4	3	3	3	3
S/G ratio	0.7	0.5	1.0	1.5	0.5	0.70
LCC linkages						
PhGlc	6.2	4.9	3.9	1.2	1.7	1.6
BE	7.1	2.2	2.8	1.5	1.8	1.1
Total	13.3	7.1	6.7	2.7	3.5	2.7

**Table 2:** The abbreviations of different AHL from different fractionation conditions.

Raw Material	Wheat Straw			Switchgrass		
Fractionation	—	M40T80 t100	M60T110 t60		M40T80 t100	M60T110 t60
Lignin samples	MWL-W	L-M40W	L-M60W	MWL-S	L-M40S	L-M60S
Delignification	-	35.1	58.2		33.8	57.4

## Data Availability

Data will be made available upon request.

## Supplementary Materials

The following supporting information can be downloaded at: https://www.mdpi.com/article/10.3390/ijms25179257/s1.
