# Supplementary material for: The Enhancement Origin of Antioxidant Property of Carboxylated Lignin Isolated from Herbaceous Biomass Using the Maleic Acid Hydrotropic Fractionation
Source: Int J Mol Sci. 2024 Aug 27;25(17):9257. doi: 10.3390/ijms25179257 (PMC11394719; doi:10.3390/ijms25179257)
Supplement: Supplementary file 1 [file ijms-25-09257-s001.zip › ijms-3126166-supplementary.pdf]

Supplementary Data

The Enhancement Origin of Antioxidant Property of Carboxylated Lignin Isolated from Herbaceous Biomass Using the Maleic Acid Hydrotropic Fractionation

Chen Su [1,2], Xiu Wang [3,*], Yongjun Deng [2], Douyong Min [1], Guigan Fang [2] and Chen Huang [1,2,*]

[1] Guangxi Key Laboratory of Clean Pulp & Papermaking and Pollution Control, College of Light Industry and Food Engineering, Guangxi University, Nanning 530004, China

[2] Jiangsu Co-Innovation Center of Efficient Processing and Utilization of Forest Resources, Institute of Chemical Industry and Forest Products, Chinese Academy of Forestry, Nanjing 210042, China

[3] Key Laboratory of Polymer Chemistry and Physics of Ministry of Education, School of Materials Science and Engineering, Peking University, Beijing 100871, China

[*] Correspondence: wangxiu@pku.edu.cn (X.W.); huangchen3127@njfu.edu.cn (C.H.)

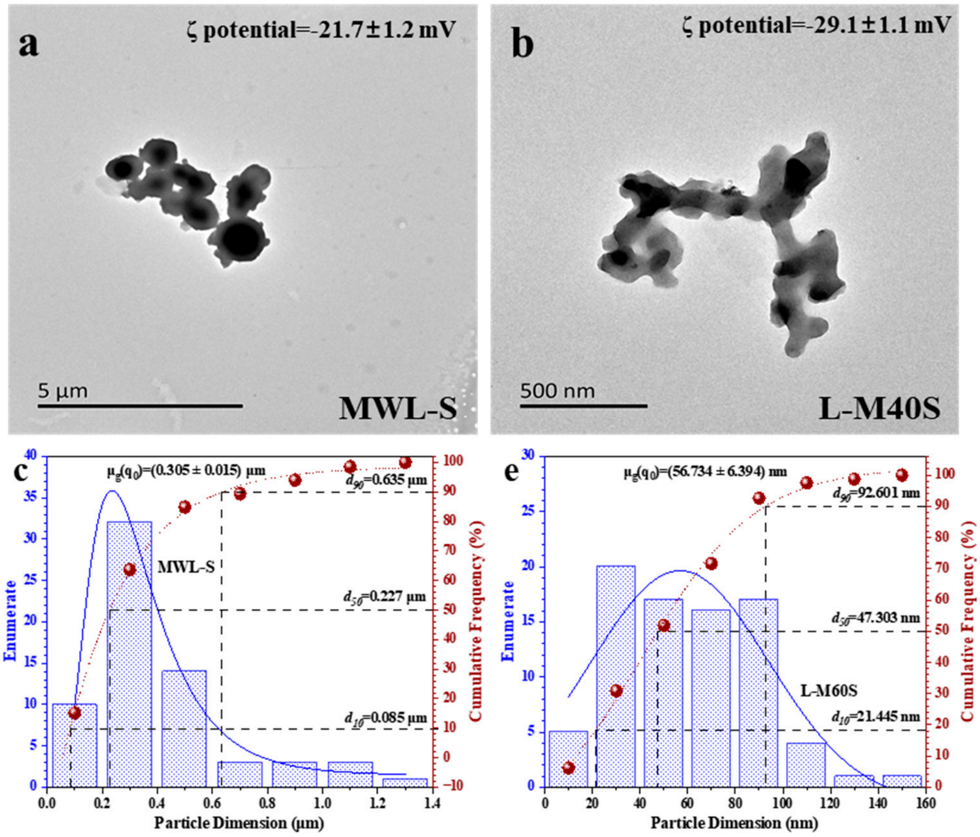

Figure S1. TEM images of MWL and AHLs, (a) MWL-S and (b) L-M40S, Particle size distribution, particle geometric mean, and ζ potential of MWL and AHLs: (c) MWL-S and (d) L-M40S。

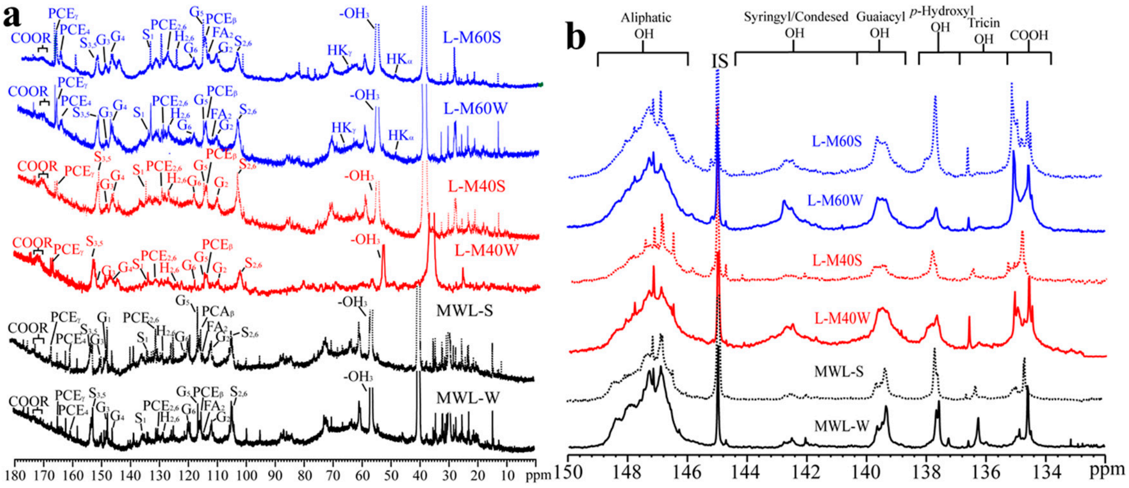

Figure S2. (a) Quantitative ^{13}C NMR and (b) ^{31}P NMR spectra of AHLs and MWLs.

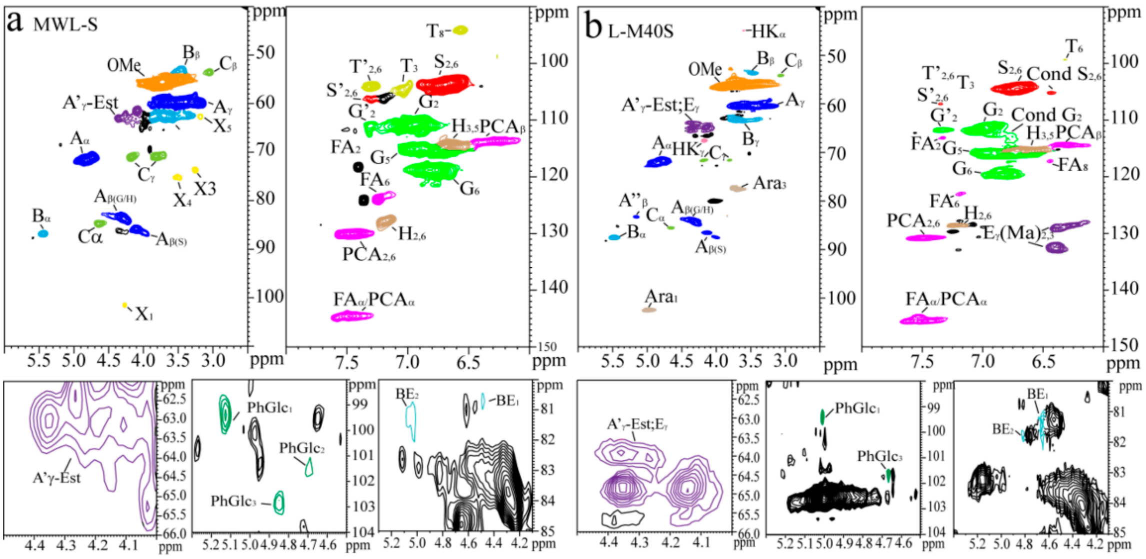

Figure S3. ^{1}H-^{13}C 2D HSQC NMR spectra of (a) MWL-S and (b) L-M40S.

Table S1. Assignment of main lignin ^{13}C-^{1}H cross-signals in the 2D HSQC spectra of MWLs and AHLs.

Labels	δ_C/δ_H (ppm)	Assignment
Lignin structures		
HKα	44.5/3.70	C_α-H_α in Hibbert's ketone (HK)
Cβ	53.7/3.46	C_β-H_β in phenylcoumarane substructures (C)
Bβ	54.1/3.06	C_β-H_β in resinol substructures (B)
OMe	56.2/3.74	C-H in methoxyls
A_γ	60.5/3.61	C_γ-H_γ in β-O-4' substructures (A)
B_γ	63.4/3.70	C_γ-H_γ in phenylcoumaran substructures (C)
E_γ(MA) $_\gamma$	63.5/3.83 63.5/4.30	C_γ-H_γ in β-O-4'l esterified with MA
A'_γ-Est	64.8/4.36	C_γ-H_γ in γ-acylated β-O-4 substructures (A')
HKγ	67.1/4.2	C_γ-H_γ in Hibbert's ketone (HK)
C_γ	71.7/4.18 71.7/3.85	C_γ-H_γ in resinol substructures (B)
A_α	72.27/4.82	C_α-H_α in β-O-4' substructures (A)
A''_β	83.3/5.16	C_β-H_β in β-O-4' substructures with *Ca*=O groups (A")
$A_{\beta(G/H)}$	84.1/4.32	C_β-H_β in β-O-4' substructures linked to G and H units (A)
Cα	85.6//4.67	C_α-H_α in phenylcoumaran substructures (C)

$A_{\beta(S)}$	86.7/4.12 87.4/4.02	C_β-H_β in β-O-4' substructures linked to S units (A)
B_α	87.6/5.46	C_α-H_α in resinol substructures (B)
T_8	94.8/6.59	C_8-H_8 in tricin (T)
T_6	99.4/6.23	C_6-H_6 in tricin (T)
$T'_{2,6}$	104.7/7.33	C_2-H_2 and C_6-H_6 in tricin (T')
T_3	105.3/7.04	C_3-H_3 in tricin (T)
$S_{2,6}$	105.5/6.71	C_2-H_2 and C_6-H_6 in etherified syringyl units (S)
Cond $S'_{2,6}$	106.3/6.43	C_2-H_2 and C_6-H_6 in condensated syringyl units (S)
$S'_{2,6}$	107.0/7.34	C_2-H_2 and C_6-H_6 in syringyl units with C*a*=O groups (S')
G_2	111.6/7.01	C_2-H_2 in guaiacyl units (G)
G'_2	111.7/7.32	C_2-H_2 in guaiacyl units with C*a*=O groups (G')
Cond G_2	112.1/6.79	C_2-H_2 in in condensated guaiacyl units (S)
FA_2	113.1/7.33	C_2-H_2 in ferulate (FA)
PCA_β	114.3/6.31	C_8-H_8 in *p*-coumarate (PCA)
$H_{3,5}$	115.2/6.70	$C_{3,5}$-$H_{3,5}$ in *p*-hydroxyphenyl units (H)
G_5	116.2/6.79	C_5-H_5 in guaiacyl units (G)
G_6	119.6/6.81	C_6-H_6 in guaiacyl units (G)
FA_6	123.1/7.18	C_6-H_6 in ferulate (FA)
J_β	126.8/6.76	C_β-H_β in cinnamaldehyde end groups (J)
$H_{2,6}$	128.5/7.23	$C_{2,6}$-$H_{2,6}$ in *p*-hydroxyphenyl units (H)
E_γ(MA)2,3	128.6/6.23 132.6/6.39	$C_{2,3}$-$H_{2,3}$ in maleic acid esterified with S or G unit lignin at γ-OH
$PCA_{2,6}$	130.7/7.48	C_2-H_2 and C_6-H_6 in *p*-coumarate (PCA)
FA_α/PCA_α	145.1/7.49	C_7-H_7 in ferulate (FA) and *p*-coumarate (PCA)
Carbohydrate		
X_5	63.1/3.22	C_5-H_5 in β-D-xylopyranoside (X)
X_2	73.1/3.06	C_2-H_2 in β-D-xylopyranoside (X)
X_3	74.6/3.28	C_3-H_3 in β-D-xylopyranoside (X)
X_4	75.9/3.52	C_4-H_4 in β-D-xylopyranoside (X)
U_4	76.9/3.13	C_4-H_4 in 4-O-methyl-α-D-glucuronic acid (U)
Ara_3	77.6/3.69	C_3-H_3 in arabinofuranoside (Ara)
Ara_2	83.0/3.76	C_2-H_2 in arabinofuranoside (Ara)
αX_1	92.9/4.89	C_1-H_1 in (1→4)-α-D-xylopyranoside
U_1	96.9/5.07	C_1-H_1 in 4-O-methyl-a-D-glucuronic acid (U)
βX_1	99.0/4.26	C_1-H_1 in (1→4)-β-D-xylopyranoside
X_1	102.3/4.27	C_1-H_1 in β-D-xylopyranoside (X)

Ara_1	102.5/4.98	C_2-H_2 in arabinofuranoside (Ara)
LCC linkages		
BE_1	81-80/ 4.7-4.5	C_α-H_α in benzyl ether (secondary OH) linkages
BE_2	81-80/5.1-4.9	C_α-H_α in benzyl ether (primary OH) linkages
PhGlc	103-88/5.20-4.65	C-H in phenyl glycoside linkages
